# Reliability of a digital system for models measurements in BBO grading: A pilot study

**DOI:** 10.1590/2177-6709.27.1.e2219388.oar

**Published:** 2022-02-28

**Authors:** Paula Martins Bravo MIRANDA, Luciana Quintanilha Pires FERNANDES, Manuel Gustavo Chavez SEVILLANO, Felipe de Assis Ribeiro CARVALHO, Jonas CAPELLI

**Affiliations:** 1 Universidade Federal Fluminense, Mestrado em Ortodontia (Niterói/RJ, Brazil).; 2 Universidade do Estado do Rio de Janeiro, Doutorado em Ortodontia (Rio de Janeiro/RJ, Brazil).

**Keywords:** Orthodontics, Dental occlusion, Dental models

## Abstract

**Introduction:**

Currently, no method is considered effective for the evaluation of digital models in the Certification Examination of the Brazilian Board of Orthodontics (BBO), considering the parameters of the currently used manual method.

**Objective:**

Thus, the aim of this study is to verify the reliability of an evaluation method for digital models that could be used in the BBO exam, compared to the gold standard.

**Methods:**

Measurements were performed by five previously calibrated examiners. Samples of ten sets of plaster models of the final phase of orthodontic treatment were measured using a manual method (Objective Grading System, OGS). These models were digitized using a 3D scanner and exported to Geomagic Qualify software, in which the measurements were made with the proposed digital method. These measurements were repeated using five models, after fifteen days. The intra-examiner performance with this method was analyzed with a paired *t*-test, whereas the inter-examiner analysis was carried out with analysis of variance and Tukey’s test. To compare the manual and digital methods, a paired *t*-test and Pearson’s correlation analysis were performed.

**Results:**

A statistically significant difference was found. The results showed that, when compared to the manual method, the digital method was effective in measuring the OGS in four of the seven variables studied: Marginal Ridge, Overjet, Occlusal Contact, and Interproximal Contact. The variables Alignment, BL inclination, and Occlusal Relationship showed a great amount of dispersion in the findings.

**Conclusion:**

Further studies are needed to develop an adequate digital methodology that can be used for all OGS variables.

## RESUMO

**Introdução:**

Ainda não há um método considerado eficaz para análise dos modelos digitais no exame do Board Brasileiro de Ortodontia (BBO), considerando-se os parâmetros do método manual atual.

**Objetivo:**

Assim, o presente estudo objetiva verificar a confiabilidade de um método de avaliação em modelos digitais para o exame do BBO, comparando com o padrão-ouro.

**Métodos:**

As medições foram realizadas por 5 examinadores, previamente calibrados. A amostra de 10 pares de modelos de gesso da fase final do tratamento ortodôntico foi medida no método manual (Sistema Objetivo de Avaliação, SOA). Os modelos foram digitalizados por meio de um *scanner* 3D e exportados para o *software* Geomagic Qualify, onde foram feitas as medidas no método digital proposto. As medidas foram refeitas em 5 modelos após 15 dias. A análise intraexaminador desse método foi realizada por meio do teste *t* pareado; já a interexaminadores, feita com ANOVA e teste de Tukey, sendo encontrada diferença estatisticamente significativa. Para a comparação dos métodos manual e digital, foram utilizados o teste *t* pareado e a correlação de Pearson.

**Resultados:**

Uma diferença estatisticamente significativa foi encontrada. Os resultados mostraram que, comparada ao método manual, a metodologia digital mostrou-se eficaz para medição do SOA em quatro das sete variáveis estudadas: Margem interproximal, Sobressaliência, Contato oclusal e Contato interproximal. As variáveis Alinhamento, Inclinação V-L e Relação oclusal mostraram muita dispersão nos achados.

**Conclusão:**

Mais estudos são necessários para o desenvolvimento de uma metodologia digital adequada em todas as variáveis do SOA.

## INTRODUCTION

The Brazilian Board of Orthodontics and Facial Orthopedics (BBO) is an entity that promotes clinical excellence within the specialty of orthodontics. During the certification process, a candidate presents the results from six clinical cases after orthodontic treatment is completed. The plaster models are evaluated under the Objective Grading System (OGS), which is used to judge the orthodontic case using a metric system developed by the American Board of Orthodontics (ABO).^1^ ^,^ ^2^ ^,^ ^3^ Also, the ABO system is considered an excellent way to self-assess cases treated in private orthodontists’ offices.^4^

Due to the increasing demand for digital models, OrthoCAD has developed a tool ^7^ to enable the inclusion of digital models in the final clinical case assessment performed in the ABO candidate examination.^8^ However, some studies evaluating this feature have determined that the tool cannot replace manual measurements in plaster models.^5^ ^,^ ^6^ ^,^ ^9^

With the emergence of scanning and digital modeling, there is a growing interest regarding the accuracy of the measures obtained from digital models, as compared to the conventionally used plaster models.^5^ No digital method was considered efficient after evaluating every item from the OGS.^6^

Considering the above-mentioned concerns, this study aimed to determine the accuracy of a digital measurement system under the OGS, compared to the conventional manual approach. 

## MATERIAL AND METHODS

Five examiners were designated, who were all specialists in orthodontics. Before starting this study, the examiners were calibrated by a BBO board member, and five different sets of study models were evaluated by them. Subsequently, ten sets of plaster models from patients who attended the Orthodontics Specialization Clinic of the State University of Rio de Janeiro (Brazil) for orthodontic treatment were selected to this study sample. Inclusion criteria were that the plaster models needed to be in good condition and the models needed to be from patients with finished orthodontic treatment that presented complete permanent dentition, except for the third molars. 

At first, the sample was measured with the manual method using the ABO measuring gauge (OGS). Afterwards, for the digital evaluation method, the models were digitized using a 3D scanner (Maestro 3D Dental Scanner - AGE Solutions, Potedera, Italy). After the images were captured, they were stored in STL open formats and exported to Geomagic Qualify 2013 software (Raindrop Geomagic, Inc., Cary, NC, USA), where the proposed digital measurement methodology was employed. An interval of 15 days was considered suitable to repeat the method in 5 sets of models of the sample, to obtain inter-examiners and intra-examiner comparisons. Lastly, the measurements of the manual and digital methods were compared to evaluate the reliability of the proposed digital method.

### PROPOSED DIGITAL METHOD

The proposed digital methodology used the same reference points that are applied to the manual evaluation system. Instead of using the ABO measuring gauge that is applied on plaster models, the Geomagic Qualify software was employed to take the measurements on the digital models. Therefore, when an alteration was detected on the ideal finalization pattern, points were placed on the sites where the ABO measuring gauge would be, and the software showed the distance between those points in the three axes (vertical, transversal, and anterior-posterior), as well as it showed the total distance. The axis of interest depended on the OGS evaluated item, which was determined by this method.

**Figure 1:**
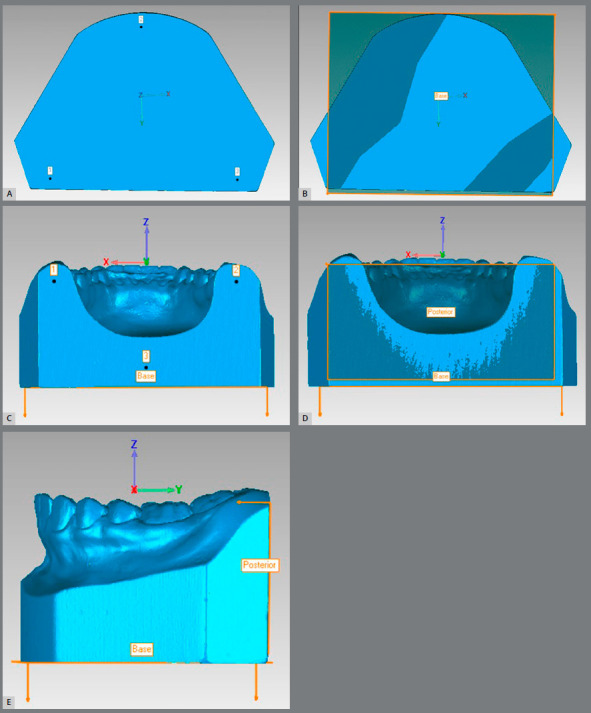
A) Positioning of the points to create the plane Base. B) Plane Base created. C) Positioning of points to create the plane Posterior. D) Plane Posterior created. E) Planes aligned with the model and the axes created according to them. Y- and X-axes are present in the Base plane, and Z- and X-axes are present in the Posterior plane.

Initially, for each model, three axes were created: the Y (anterior-posterior) axis, X (transverse) axis, and Z (vertical) axis. Two reference planes were created in the lower part of the model, termed the Base and the Posterior, which corresponded to the base and posterior surfaces of the model, respectively, through the 3-point markings (Figs 1A-1D). Thus, this newly created coordinate system was made to conform to the positioning of the digital model (Fig 1E).

**Figure 2:**
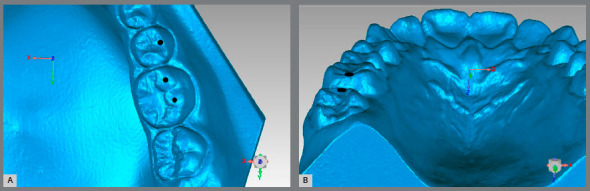
A) Measurement of a misalignment. B) Incorrect measure of the Marginal Ridge.

**Figure 3:**
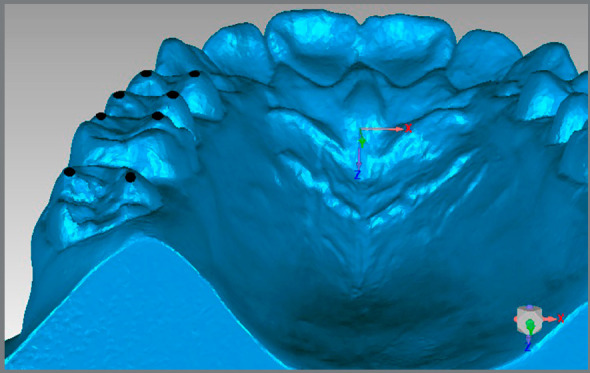
Measurement of the Buccolingual Inclination for the upper teeth.

**Figure 4:**
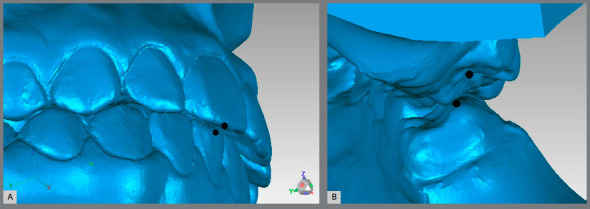
A) Measurement of the anterior incorrect Overjet. B) Measurement of the posterior incorrect Overjet.

**Figure 5:**
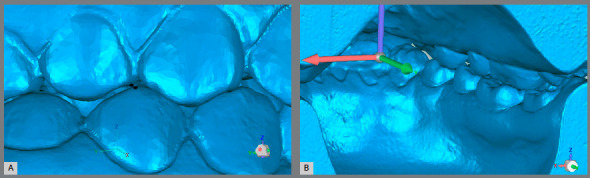
A) Measurement of the incorrect Occlusal Contact relationship, from a lingual view. B) Lingual view of another model showing the correct Occlusal Contact.

**Figure 6:**
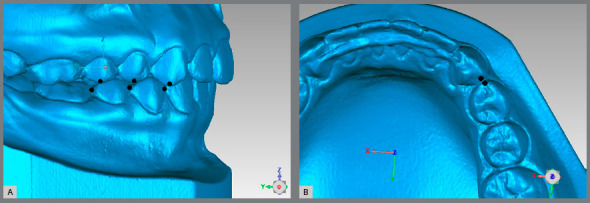
A) Measurement of the Occlusal Relationship. B) Measurement of the Interproximal Contact.

For the Alignment variable, points were placed on the teeth that were considered to be misaligned. For the posterior teeth, the transverse X-axis was observed, and for the anterior teeth, the total distance was generated (Fig 2A). For the Marginal Ridge variable, points were placed on the ridges with a difference in height, and the distance generated on the vertical Z-axis was observed (Fig 2B). In the Buccolingual Inclination (BL inclination), as seen through the height difference of the buccal and palatal cusps, the vertical Z-axis was observed (Fig 3). For the Overjet, when there was a lack of contact in the anterior teeth, points were placed in the incisal edge of the upper teeth and in the buccal face of the lower teeth, and the total distance generated was considered (Fig 4A). For the posterior teeth, the distance where the teeth should be occluding, if not in the ideal position, was observed, and the transverse X-axis was the focus of interest (Fig 4B). The Occlusal Contact variable was observed with the models in occlusion and was evaluated only with the posterior teeth. If there was no contact, the distance from the place where the teeth in question should occlude was measured, and the vertical Z-axis was observed (Figs 5A and 5B). In the Occlusal Relation, the occlusion relationship of the canines and posterior teeth was observed, and measurements were made between the upper cusps and the point where they should occlude with the lower teeth, and the distance in the anteroposterior Y-axis was observed (Fig 6A). Finally, in the Interproximal Contact, if there was no contact between any teeth, the points were placed on the distal and mesial area of the teeth in question, and the distance between them was measured. For the anterior teeth, the total distance was considered, and for the posterior teeth, the anteroposterior Y-axis was considered (Fig 6B).

### STATISTICAL ANALYSIS

Statistical analysis was performed using SPSS software (version 22.0; SPSS, Chicago, IL, USA). A *p*-value < 0.05 was considered significant. A Shapiro-Wilk test was applied to verify the normality of the sample.

In the digital method, a paired *t*-test was used to check the reproducibility of the scores, and the null hypothesis was that there was no correlation between the measurements made the first time (T_1_) and the second time (T_2_). A strong correlation was r > 0.7, a moderate correlation was r between 0.7 and 0.3, and a weak correlation was r < 0.3. All three conditions were considered. To analyze inter-examiners reproducibility, an analysis of variance (ANOVA) was used, and the null hypothesis was that there was no difference between the groups. In case of a significant difference, Tukey’s test was applied to the variable in question.

In the analysis that compared the manual and digital methods, two tests were applied for each evaluated variable: a paired t-test to evaluate the scores between the methods and Pearson’s correlation test to verify if there was a correlation between the measurements. Correlations were classified according to the value of r, with r > 0.7 indicating a strong correlation, r between 0.7 and 0.3indicating a moderate correlation, and r < 0.3 indicating a weak correlation.

## RESULTS

The Shapiro-Wilk test showed that the data formed a normal distribution.

In the intra-examiner analysis (Table 1), the only variable that showed a strong correlation between T_1_ and T_2_ for all evaluators was Occlusal Contact. The other variables did not show a statistically significant correlation for all the evaluators, as there was always an evaluator with a moderate or weak correlation. Although the statistical test did not detect a strong correlation, the values of the scores were very close. For some variables, the program was not capable of generating p and/or r values ​​due to a constant score of 0 by the evaluator.

**Table 1:** Paired t-test for intra-examiner (Ex) evaluation of measurements in digital models.

Paired t-test						
Variable		Ex1	Ex2	Ex3	Ex4	Ex5
Alignment	T1 meas.	11.80	12.40	7.20	1.60	6.60
	T2 meas.	10.40	12.80	7.40	3.60	6.20
	*p*	0.068***	0.232**	0.046***	0.042***	0.049***
	r	0.851***	0.654**	0.884***	0.891***	0.880***
Marginal Ridge	T1 meas.	2.40	2.80	2.60	0.40	3.20
	T2 meas.	1.40	3.00	2.20	1.40	4.20
	*p*	0.898*	0.659*	0.103***	0.599*	0.111***
	r	0.080*	0.271*	0.802***	-0.320*	0.792***
BL inclination	T1 meas.	7.20	7.40	3.40	3.60	3.60
	T2 meas.	7.40	7.00	5.40	5.80	5.80
	*p*	0.081***	0.148***	0.696*	0.272**	0.272**
	r	0.831***	0.745***	-0.241*	0.613**	0.613**
Overjet	T1 meas.	1.00	1.20	1.00	0.40	1.20
	T2 meas.	0.40	0.80	0.80	0.40	2.20
	*p*	0.01***	0.028***	0.007***	0.789*	0.784*
	r	0.913***	0.919***	0.968***	0.167*	0.116*
Occlusal Contact	T1 meas.	0	0	0	0	0
	T2 meas.	0.40	0.40	0.20	0	0
	*p*	-	-	-	-	-
	r	1.0***	1.0***	1.0***	1.0***	1.0***
Occlusal Relationship	T1 meas.	4.00	8.20	0.60	0.40	4.00
	T2 meas.	4.40	6.60	0.20	0.20	4.60
	*p*	0.368*	0.010***	0.685*	0.272**	0.002***
	r	0.521*	0.958***	0.250*	0.612**	0.987***
Interproximal Contact	T1 meas.	3.60	0	1.80	0	0.40
	T2 meas.	4.80	0	2.20	0.2	0.2
	*p*	0.496**	-	0.918*	-	0.001***
	r	0.408**	1.0***	0.064*	-	1.0***

*** Strong correlation. ** Moderate correlation. * Weak correlation. - constant number, hindering the generation of values from *p* and r.

Regarding inter-examiners agreement (Table 2), the ANOVA test showed a statistically significant difference in the Alignment, BL Inclination, Occlusal Relationship, and Interproximal Contact. When it was found a significant difference in some variable, the Tukey test was applied in that variable, in order to identify which examiners showed difference. Tukey’s test showed that for Alignment, examiners 4 and 5 gave values ​​that displayed statistically significant differences from the others. For BL Inclination, there was no statistically significant difference between the examiners. For the variables Occlusal Relationship and Interproximal Contact, there were large variations among the examiners.

**Table 2:** ANOVA test to evaluate the inter-examiner evaluation of measurements in digital models.

Variable	ANOVA		Tukey’s test
	F	P	
Alignment	18.106	0.000*	Difference in evaluators 4 and 5
Marginal Ridge	0.451	0.771	
BL inclination	2.739	0.040*	No difference between evaluators
Overjet	1.980	0.114	
Occlusal Contact	0.115	0.977	
Occlusal Relationship	16.864	0.000*	Difference between almost every evaluator
Interproximal Contact	10.858	0.000*	Difference between almost every evaluator

*Statistically significant difference.

When comparing the manual and digital methods, the paired *t*-test showed statistically significant differences in the variables Alignment, BL Inclination, and Occlusal Relationship (*p* < 0.05; Table 3).

**Table 3:** Paired t-test for comparison of scores in conventional and digital methods.

Variable	Mean	S.D.	Mean S.D.	Confidence interval at 95% difference		t	t-test Significance
				Upper	Lower		
Alignment	-3.360	4.754	0.672	-2.009	-4.711	-4.997	0.000*
Marginal Ridge	0.560	2.012	0.285	1.132	-0.012	1.132	0.055
BL inclination	-1.820	2.430	0.344	-1.129	-2.511	-5.296	0.000*
Overjet	-0.266	1.850	0.262	0.266	-0.786	-0.994	0.325
Occlusal Contact	0.000	0.571	0.081	0.162	-0.162	0.000	1.000
Occlusal Relationship	-2.040	3.149	0.445	-1.145	-2.935	-4.581	0.000*
Interproximal Contact	-0.520	1.887	0.267	0.016	-1.056	-1.949	0.057

S.D. = Standard deviation. *Statistically significant difference.

Table 4 presents the Pearson correlation coefficients associated with the degree of significance. The only variable that showed no statistically significant correlation between the two methods was Alignment (*p*> 0.05). Only Occlusal Contact showed a strong correlation.

**Table 4:** Pearson’s correlation results between manual and digital measurements.

Variable	Pearson’s	
	Significance	R
Alignment	0.601*	0.076*
Marginal Ridge	<0.001***	0.516**
BL inclination	0.001***	0.544**
Overjet	0.05***	0.279*
Occlusal Contact	0.001***	0.702***
Occlusal Relationship	0.002***	0.427**
Interproximal Contact	0.001***	0.482**

*** Strong correlation. ** Moderate correlation. * Weak correlation.

Pearson’s correlation scatter plots were generated for each of the variables, where the Y-axis represents the scores made by the manual method and the X-axis represents the digital scores. From these results, it was observed that the digital method scored higher than the manual method, indicating that it is a more sensitive method. The graphs that showed the best correlations are for Marginal Ridge, BL Inclination, and Occlusal Contact.

## DISCUSSION

A study was conducted with the Geomagic Qualify software,^10^ which allows the creation of a coordinate system through three axes in space and the projection of the distance between two points, which is a feature that is not available in OrthoCAD. However, some disadvantages with the methodology were described by the authors, such as the time-consuming execution, since points were recorded for every tooth, regardless of its position. In the present study, although digital method took longer, it was not that expressive. We observed approximately 17 minutes for executing the manual method and 21 minutes for the digital (2 minutes for the creation of the coordinate system and 19 minutes for the measurements of the variables). However, the addition of five minutes to each model can make a difference to someone who is evaluating a lot of models in the certification process of the BBO.

In the present study, in intra-examiner analysis, despite the low correlations found (Table 1), the measures of the variables were similar, indicating that a larger sample could be more efficient in identifying significant differences. In addition, the digital method may need to be further calibrated, as orthodontists have less experience with this technology than with the manual approach. In the inter-examiner agreement, there was no reproducibility of the method for the variables Alignment, Occlusal Relationship, and Interproximal Contact (Table 2).

For Alignment, the correlation in Pearson’s correlation analysis could not be confirmed based on the *p*-value (Table 4), and a statistically significant difference was found only with the paired t-test (Table 3). Thus, the digital method was not compatible with the manual method in this domain. This difference may have occurred due to the angle of this variable in relation to the coordinate axis, since it does not follow the shape of the dental arches. Since the front teeth are in front of that shape, it became a challenge to measure their misalignment on one of the horizontal axes (X or Y), so the total distance was considered. However, this did not eliminate the vertical variation of the points, and if there is an angle between them, it can generate an increase in the true distance, thus causing a possible higher score. This also occurred in the posterior teeth, which continue shaping the sides of the arch, and although less curved, they were not parallel to the anteroposterior X-axis, which was used to measure the misalignments. This may have been one of the reasons for the large difference in this variable. Of three studies evaluating the OrthoCAD program, two found statistically significant differences for this variable,^5^ ^,^ ^9^ and one study proposed a method using Geomagic Qualify software.^10^ Another study, however, found consistency between the manual and non-manual digital measurements.^6^

For the Marginal Ridge, no statistically significant difference was found with the paired *t*-test (Table 3), and there was a moderate correlation in Pearson’s correlation analysis (Table 4). Therefore, it can be considered that there was a consistency in its measurement, which is a result compatible with studies evaluating OrthoCAD^5^ ^,^ ^6^ ^,^ ^9^ and a previous study that employed Geomagic Qualify.^10^

For the BL Inclination, the paired t-test showed a statistically significant difference (Table 3), although Pearson’s correlation analysis showed a moderate correlation (Table 4). This indicated that although the measurements differed regarding the methods, as one increased, the other also increased. Thus, for the application of this variable, a new scoring table is suggested, since the digital method scored higher than the manual method, but with a similar proportion. This variable showed a statistically significant difference in a study evaluating OGS in digital models by OrthoCAD.^9^ In the study with Geomagic Qualify, there was consistency between the manual and digital methods,^10^ as demonstrated in another study.^5^

For the Overjet, no statistically significant difference was found with the t-test (Table 3), but the correlation found with Pearson’s correlation analysis was weak. This variable may also have been influenced by difficulty in following the measurements with the shape of the arches. In the posterior teeth, the score was calculated from the anteroposterior X-axis, and the arch form may have interfered with the distance calculated by the program. In the anterior teeth, where the total distance was observed, the vertical variation of the points may have influenced the results. Thus, further studies are needed to assess the digital application for this variable. From the studies with the OrthoCAD program, as well as the study with Geomagic Qualify, one study found a statistically significant difference in this variable,^5^ while the other studies found consistency between the manual and digital measurements.^6^ ^,^ ^9^

For Occlusal Contact, the methodologies were considered to be comparable, as no statistically significant difference was found with the paired *t*-test (Table 3), in addition to obtaining a strong correlation with Pearson’s correlation analysis (Table 4). Of the other OrthoCAD studies, only one did not find a statistically significant difference,^9^ and the study that used Geomagic Qualify did not find a difference as well.^10^ The others found a statistically significant difference.^5^ ^,^ ^6^

For the Occlusal Relationship variable, a statistically significant difference was found with the paired *t*-test (Table 3) and a moderate correlation was found with Pearson’s correlation analysis (Table 4). Thus, as in the BL Inclination, for its application, a new scoring table is suggested, since the digital method scored higher than the manual method, but with a similar proportion. This result was similar to that found in the study using Geomagic Qualify^10^ and in a study evaluating OrthoCAD,^6^ whereas in the other two studies, no statistically significant differences were found.^5^ ^,^ ^9^

For Interproximal Contact, no statistically significant difference was found with the paired *t*-test (Table 3), and the correlation was moderate by Pearson’s correlation analysis (Table 4), indicating that it is an alternative to the digital methodology. These results concurred with the studies already cited.^5^ ^,^ ^6^ ^,^ ^9^ ^,^ ^10^

The differences found between the manual and digital methodologies should be analyzed with some considerations. Although evaluation using plaster models is considered the gold standard, this method was created for clinical purposes and has some methodological limitations. Among them is the parallax effect, which is a different assessment depending on the angle at which the observer looks at the model. This effect would be negated with digital models using the method suggested in a study where points were placed on all teeth.^10^ However, their results showed compatibility only with the BL Inclination and Occlusal Contact variables. Thus, the present study opted for an attempt to approach the manual method, making use of the observation of the models through free manipulation in the software, allowing measurements only of the wrong areas observed, as is done with the manual method. However, even with this free way of view in the method of this study, the manual method seemed more subjective, which can be reinforced by a study that evaluated the reliability and frequency of point subtraction in study models by different examiners. The results showed that some examiners were, on average, less strict than others.^11^

In addition, the examiners in this study reported the accuracy of digital measurements. In the manual method, the millimeters observed and measured with the ruler may be approximated by the human eye due to a lack of precision of the ruler. Digitally, there is no approximation, since the measurement between the two points is generated by a virtual program that provides the exact measurement between them, including measurements in micrometers. Thus, a measure of 2.152 in the software program, which could be approximated by the human eye to 2, could generate over-scoring depending on the OGS variable. To eliminate this digital acuity, for future studies, it is suggested that a scale be created by which the software shows the measurement only of the numbers present in the scale, indicating whether the measurement is closer to 2 or 2.5 mm, for example. This would approach the differences that could be perceived by the ruler. The higher digital method score found in this study is in agreement with other studies.^6^ ^,^ ^10^

Another limitation of the digital methodology is the angle of some measurements in relation to the coordinate axis, due to the dental arches form, as already mentioned in Alignment and Overjet.

Based on these results, it can be stated that orthodontics is in a transition period from plaster to digital models. Adapting to this technological advance is necessary and inevitable. Based on the findings, further studies are needed to create an appropriate digital methodology and to develop digital tools that are specific for this purpose. This would combine the strengths of the works already completed. 

## CONCLUSION

When compared to the manual approach, the digital method was effective in measuring the Objective Grading System in four of the seven variables studied: Marginal Ridge, Overjet, Occlusal Contact, and Interproximal Contact. Three variables, Alignment, BL Inclination, and Occlusal Relationship, showed greater dispersion of their values. Therefore, further studies are needed to develop an appropriate digital method for all OGS variables.
